# Internal iliac and uterine arteries Doppler ultrasound in the assessment of normotensive and chronic hypertensive pregnant women

**DOI:** 10.1038/srep03785

**Published:** 2014-01-21

**Authors:** L. Guedes-Martins, A. Cunha, J. Saraiva, R. Gaio, F. Macedo, H. Almeida

**Affiliations:** 1 Departamento de Biologia Experimental, Faculdade de Medicina da Universidade do Porto, 4200–319 Porto, Portugal; 2 IBMC-Instituto de Biologia Molecular e Celular, 4150–180 Porto, Portugal; 3 Centro Hospitalar do Porto EPE, Departamento da Mulher e da Medicina Reprodutiva, Largo Prof. Abel Salazar, 4099–001 Porto, Portugal; 4 Department of Mathematics, Faculty of Sciences of the University of Porto, Portugal; 5 CMUP-Centre of Mathematics of the University of Porto, Portugal; 6 Departamento de Medicina, Faculdade de Medicina da Universidade do Porto, 4200–319 Porto, Portugal; 7 Centro Hospitalar S. João, 4200–319 Porto, Portugal; 8 Ginecologia-Obstetrícia, Hospital-CUF Porto, 4100 180 Porto, Portugal

## Abstract

The objective of this work was to compare Doppler flows pulsatility index (PI) and resistance indexes (RI) of uterine and internal iliac arteries during pregnancy in low risk women and in those with stage-1 essential hypertension. From January 2010 and December 2012, a longitudinal and prospective study was carried out in 103 singleton uneventful pregnancies (72 low-risk pregnancies and 31 with stage 1 essential hypertension)at the 1^st^, 2^nd^ and 3^rd^ trimesters. Multiple linear regression models, fitted using generalized least squares and whose errors were allowed to be correlated and/or have unequal variances, were employed; a model for the relative differences of both arteries impedance was utilized. In both groups, uterine artery PI and RI exhibited a gestational age related decreasing trend whereas internal iliac artery PI and RI increased. The model testing the hemodynamic adaptation in women with and without hypertension showed similar trend. Irrespective of blood pressure conditions, the internal iliac artery resistance pattern contrasts with the capacitance pattern of its immediate pelvic division, suggesting a pregnancy-related regulatory mechanism in the pelvic circulation.

Shortly after the establishment of pregnancy, the maternal circulation undergoes a substantial change to meet the increasing demands of the growing uterus and foetus.

Noteworthy, there is a decrease in total peripheral resistance until midpregnancy^1^,^2^ and a >40% increase of cardiac output measured at the aortic valve^2^ that results from enhanced heart rate, a slight increment in the aortic valve area and an increased blood flow velocity across the valve^1^,^2^. This change is reflected in the pelvic circulation, where volume flow and mean velocity also increase in common iliac arteries^3^. However, past this point, a notorious difference is noticed.

In fact, while in the external iliac artery, that feeds the lower limb, mean velocity and volume flow are reduced along the pregnancy, in contrast, the uterine artery (UtA) exhibits a progressive and persistent increase in volume flow and mean velocity^3^,^4^. This pregnancy related redistribution of blood to the pelvis is particularly important as the uterine artery provides most of the blood to the uterus and is thus critical to the continuous and adequate foetal nourishment.

In the mid-1990s, the procedures employed in the acquisition of those data, many of them based in velocity measurement, were replaced in part by indices relying on the computerized analysis of Doppler ultrasound frequency spectrum. Such indices include the pulsatility index (PI) and the resistance index (RI), both derived from blood velocity measurements at specific points of the systolic/diastolic cycle. As they are easy to obtain, do not require cumbersome adjustments and are more objective, they have been widely applied in the assessment of the uteroplacental circulation in normal pregnancies^4^ and also when they are complicated by pathological conditions as hypertensive disorders^5^,^6^,^7^. In this setting, the uterine artery became an important target in the application of those procedures^8^,^9^,^10^,^11^,^12^,^13^,^14^,^15^.

The enhanced blood volume that is transported by the uterine arteries along the pregnancy is associated with a reduced impedance of flow consequent to the impressive structural changes that take place at the placental bed. In normal pregnancy, placental trophoblastic cells migrate across the decidua, invade the inner third of the myometrium and replace most of the muscular and endothelial cells of the maternal spiral arteries, rendering them low impedance, high capacitance vessels that optimize the delivery of oxygen and nutrients to the foetus. That change is reflected in the uterine artery flow velocity as measured by Doppler ultrasound spectrum^5^,^6^,^13^.

It was noticed that in the non-pregnant state there is a rapid rise and sudden fall in flow velocity during systole and a “notch” in early diastole^16^, a property that reflects an high impedance vessel. As the pregnancy evolves normally from 8 weeks onwards, a progressive increase in uterine artery compliance is noticed, which continues through 26 weeks' gestation, albeit at a lesser extent^16^, during which the «notch» is smoothed and lost.

However, when the trophoblast invasion is defective, an enhanced placental vascular resistance is likely to occur as evidenced in abnormal Doppler ultrasound spectrum of the uterine vessels, indicating women at risk for serious pregnancy disorders like preeclampsia^5^,^12^,^13^,^14^. In fact, abnormal uterine artery blood flow employing Doppler ultrasound assessment, at both the first and second trimesters, was shown to associate with subsequent perinatal complications^17^,^18^,^19^,^20^,^21^ and some studies even referred the analysis of Doppler wave variations as a means to assess the potential benefits of therapeutic interventions^20^,^21^,^22^,^23^.

The results of the hemodynamic studies of the UtA made so far are evidence of its close relation to the changes taking place in the growing uterus. Such studies contrast with the scarcity of data concerning other pelvic arteries as was pointed out^24^. In fact, it is noteworthy that, while the UtA has been a subject of extensive research, its predecessor, the internal iliac artery (IIA), has not. This is unexpected because it is an easily accessible artery, both in pregnant and non-pregnant women, and is also the first artery entering the pelvis. Thus, it was hypothesized that such unique property would render the IIA an important means for the understanding of the pelvic circulation along the pregnancy.

For this purpose, the most immediate approach would be a longitudinal study of uneventful pregnancies in healthy women. Yet, it was also reasoned that additional information on artery performance along the pregnancy would be provided by a parallel study in women having long term, stable, essential hypertension, a prevalent condition and also a known risk factor for serious disorders of the pregnancy^25^,^26^,^27^,^28^,^29^.

On account of those considerations, particularly the lack of knowledge on the internal iliac artery hemodynamics, in contrast with the larger knowledge on the uterine artery, it was purposed to compare blood flow of both at several time points throughout the pregnancy, employing Doppler ultrasound spectrum analysis.

## Results

The main characteristics and pregnancy outcomes of the 103 women are depicted in Table 1. Their age ranged from 17 to 43 years old, 69% of them were less than 34 years old and a similar proportion (74%) had not been educated beyond the secondary level (12 years at most), perhaps because our hospital covers an area with important socio-economic difficulties. For 52% of the women, this was their first pregnancy. A total of 54% of the population studied had a BMI between 18 and 24 Kg/m^2^ at the first appointment. The average time of the ultrasound evaluation for the three trimesters was 13.04 weeks (range: 11.43–14.14), 20.73 weeks (range: 19.14–23.71) and 30.46 weeks (range: 28.71–33). They all delivered at term^30^,^31^ with an average at 38.9 weeks (range: 37.14–41).

Regarding NT and HT groups, statistically significant differences were found for BMI (higher classes predominantly in the HT group) and age (older classes predominantly in the HT group).

**Figure 1:**
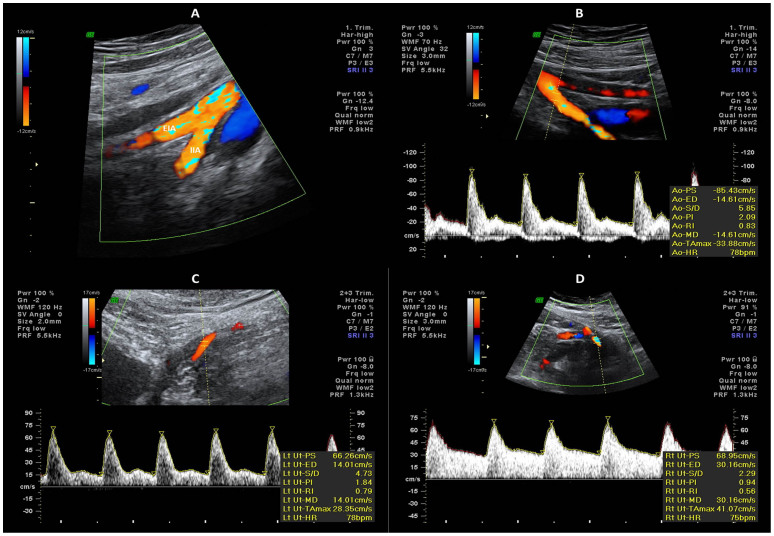
(A) External iliac (EIA) and internal iliac (IIA) arteries color flow mapping; (B,C,D) Doppler waveform of internal iliac and uterine arteries: notice the biphasic flow of internal iliac artery (B), and uterine artery notch presence (C) or absence (D).

The presence of uterine artery notching declined along pregnancy, from 48% to 5%, as expected (Table 2). In the first trimester, significant differences for the presence of bilateral notching were not observed; however, in the second and third trimesters, a clear majority of women did not exhibit bilateral notching (Figure 1).

The means and standard deviations of PIs and RIs for both arteries, according to the different trimesters, the normotensive and the hypertensive groups, are displayed in Table 3.

### Multivariate analysis and predictions

The (net) effect of the gestational trimesters on the mean values of the indexes were considered merely indicative; therefore, multivariate analyses had to be performed, by adjusting that effect to potential confounders and taking the study design into consideration. As the difference between the average evaluation time in trimester 2 and that in trimester 1 was approximately equal to the difference between the average evaluation time in trimester 3 and that in trimester 2 (more precisely, the latter is 1.3 times the former), the multivariate regression model considered the variable *gestational trimester* as continuous. The correspondent model was described in the *Statistical Analysis* section; estimates of the coefficients and respective 95% confidence intervals are presented in Table 4. Statistically significant time curves were obtained for the different combinations of indexes, vessels, hypertension and presence of notching status. The residual standard error was estimated at 0.066 (degrees of freedom: 1236 total, 1220 residual) and the Bayesian Information Criterion^32^ was of −956. The parameter for the first order autoregressive time structure was estimated at 0.350, while the variance of the pulsatility index was estimated to be 6.342 times greater than the variance of the resistance index.

Known confounding variables such as maternal age, smoking habits, body mass index and the parity were also taken into account in the analysis; as they were not shown to be statistically significant, they were not considered in the final model.

For each index, a model for the proportion of uterine artery changes relative to the IIA values was also considered. The significance of the estimated coefficients (Table 5) shows that hypertension alone was not a significant predictor in the regression. Its presence in the model is due to the significant interaction between hypertension status and index. All remaining variables proved to have a significant effect on the response, including interaction effects of gestational time and index, and of gestational time and status for the presence of notching. The residual standard error was estimated at 0.099 (degrees of freedom: 618 total, 610 residual) and the Bayesian Information Criterion was of −978. The parameter for the first order autoregressive time structure was estimated at 0.403, while the variance of the pulsatility index was estimated to be 1.321 times greater than the variance of the resistance index.

### Pulsatility and Resistance indices

**Figure 2:**
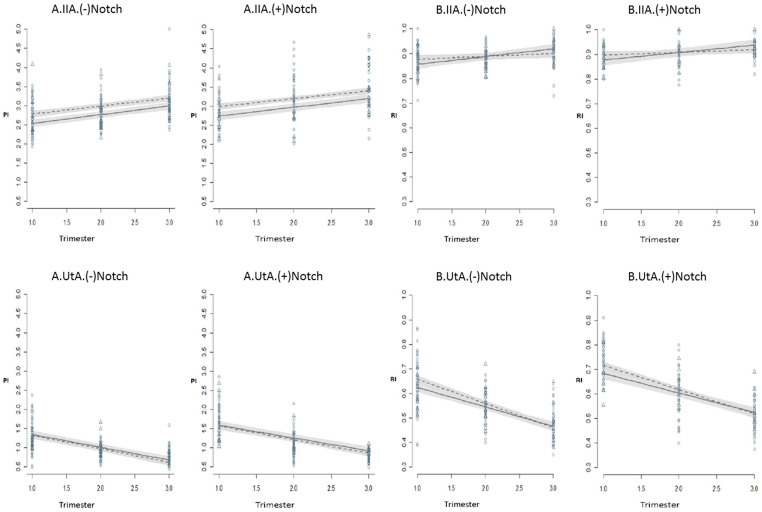
Predicted mean PI (A) and RI (B) indices for IIA and UtA during pregnancy in normotensive (dashed line, circles) and hypertensive (solid line, triangles) women in the condition of UtA notch absence (left charts) or presence (right charts). Gray bars: 95% confidence intervals for the prediction. PI and RI: Pulsatility and resistance indices; IIA and UtA: Internal Iliac and uterine arteries.

The predicted mean indexes (PI and RI) and their 95% confidence intervals for IIA and UtA during pregnancy in HT and NT pregnant women are on display in Figure 2. In both groups regardless of the absence/presence of notch or hypertension, the simple inspection of the chart shows that UtA-PI and UtA-RI follow a significant downward trend along the gestational age whereas the IIA-PI and IIA-RI show a regular upward tendency.

The PI value of the IIA over time is significantly higher in normotensive women along all trimesters, in contrast with the UtA situation (Figure 2A). The presence of notching does not change the trend and only appears to level the PI up, compared to the condition of absent notch. The PI level at the start is also significantly different when both arteries are compared, as it is higher for the IIA and lower for the UtA, and they diverge progressively along the gestational age.

The inspection of RIs of both IIA and UtA shows that, similarly to the PIs, there is a significant upward and downward trend respectively. Again, notching does not change the trend and only appears to add RI units to the absent notch condition.

In contrast with the PIs, which evidence parallel slopes when NT and HT groups are compared, the RIs of the HT condition point to higher (in the case of the IIA), or smaller (actually convergent in the case of UtA) values at the 3^rd^ trimester when compared to the normotensive condition (Figure 2B). Similarly, the RI at the start (first trimester) is significantly higher for the IIA than for the UtA, and is independent of the hypertensive condition.

### Relative change (IIA index value – UtA index value)/(IIA index value)

**Figure 3:**
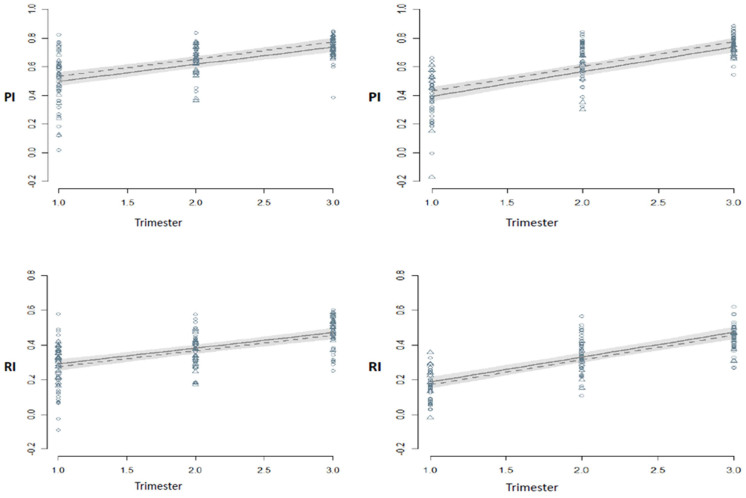
Relative changes of IIA to UtA PI (top panels) and RI (bottom panels) along the pregnancy in normotensive (dashed line, circles) and hypertensive (solid line, triangles) women in the condition of UtA notch absence (left charts) or presence (right charts). Gray bars: 95% confidence intervals for the prediction. Notice the different y-axis scale for PI and RI. PI and RI: Pulsatility and resistance indices; IIA and UtA: Internal Iliac and uterine arteries.

The predicted and observed index proportions (*i.e.*, relative changes) along the pregnancy are depicted in Figure 3. In both indexes, regardless of the state of notch and blood pressure, the proportion undergoes a significant increase over time. The statistical significance of the interaction terms between time and index type, and time and presence/absence of notch, in Table 5, show that the growth rate is significantly influenced by the presence of notch and the type of index. For PI and in women with (+) Notch, the growth rate is the highest. The isolated effect of the hypertensive state on the proportions is not significant but the existence of a significant interaction with the index shows that in the first trimester, mean PI proportion values were lower in hypertensive women compared to normotensive women; the growth rate in the succeeding times was similar. At the beginning of the gestation, the expected relative change for pregnant women with (+) Notch is significantly lower than that in pregnant (−) Notch, for both indices. Over time, mean proportion values in pregnant women with (+) Notch grow faster than on (−) Notch pregnant regardless of the values of other variables – Figure 3.

## Discussion

The great obstetrical syndromes, as preeclampsia (PE), intra-uterine growth restriction (IUGR), preterm labour and *abruptio placentae* associate to placentation disorders that result from local abnormal vascular remodelling^33^. While effective interventions to prevent such late pregnancy complications are necessary, it is also required that early, reliable, diagnostic or predictive tests become available to support the decisions to undertake such interventions.

On account of the crucial role played by the vascular network, there is currently a wide recognition of Doppler ultrasound studies importance in pregnancy evaluation and, indeed, its application in foetal and mother's pelvic circulation assessment have been of unquestionable interest^13^. Consequent to the widespread availability of equipment, a large number of studies devoted to the analysis of uterine artery blood flow patterns were published, employing the resistance index (RI) and the pulsatility index (PI). These are end-arteriolar vascular impedance indices^34^ and therefore, provide qualitative and quantitative data pertaining to local blood flow velocity and vascular resistance.

With the purpose to investigate the uteroplacental circulation^19^,^35^, emphasis has been put on the study of uterine arteries in the course of the normal pregnancy or its association with the risk for placentation disorders^20^. There is a general agreement that a progressive decline in uterine artery vascular resistance accompanies the normal pregnancy^5^,^6^,^7^,^8^,^12^,^13^,^14^,^16^,^18^. The regularity of the trend led to the establishment of reference ranges for uterine artery mean PI, covering the pregnancy from 11 to 41 weeks of gestation^6^. They show that along the normal course of the pregnancy, there is a regular decrement of PI, indicating that the uterine arteries conversion from resistance vessels (narrow bore) to high capacity vessels (larger bore), is most important to meet the increased demands of blood by the growing foetus^8^,^11^,^34^. In contrast, the observation of high resistance patterns in the uterine arteries of pregnant women was associated with local reduction of blood supply, higher incidence of preeclampsia^5^,^7^,^8^, intrauterine growth retardation^11^,^19^,^35^ and more unfavourable perinatal outcomes^11^,^35^.

Despite the wealth of data in uterine arteries, there is a lack in the study of other important vessels as the internal iliac artery, a thick, 3–4 cm long division of the common iliac artery and the main artery entering the pelvis, whose anterior division gives off the uterine artery.

As such characteristics are relevant in the context of pelvic circulation assessment, a longitudinal collection of PIs and RIs of IIA and UtA was made in every trimester of uneventful pregnancies of healthy women. As expected, mean UtA-PI and UtA-RI values showed statistically significant progressive decreases from the 1^st^ to the 3^rd^ trimester of pregnancy, a necessary adaptation that is consistent with the foetal trophoblast cell invasion of the walls of spiral arteries (the arteriolar tips of the uterine artery successive branching), the process regarded as underlying the uterine arteries change from resistance into capacitance vessels.

In notorious contrast with the uterine artery, the IIA-PI and IIA-RI evidenced a remarkable increase in the same period. To the best of our knowledge, this is the first time such findings are reported.

This change is quite interesting because, although the internal iliac artery immediately precedes the uterine artery, it exhibits an entirely distinct performance. In fact, instead of becoming a capacitance vessel, the internal iliac arteries behaved much like resistance vessels and their progressive RI and PI increase reflect an enhanced blood velocity, similarly to the previous observation in the common iliac artery^3^.

**Figure 4:**
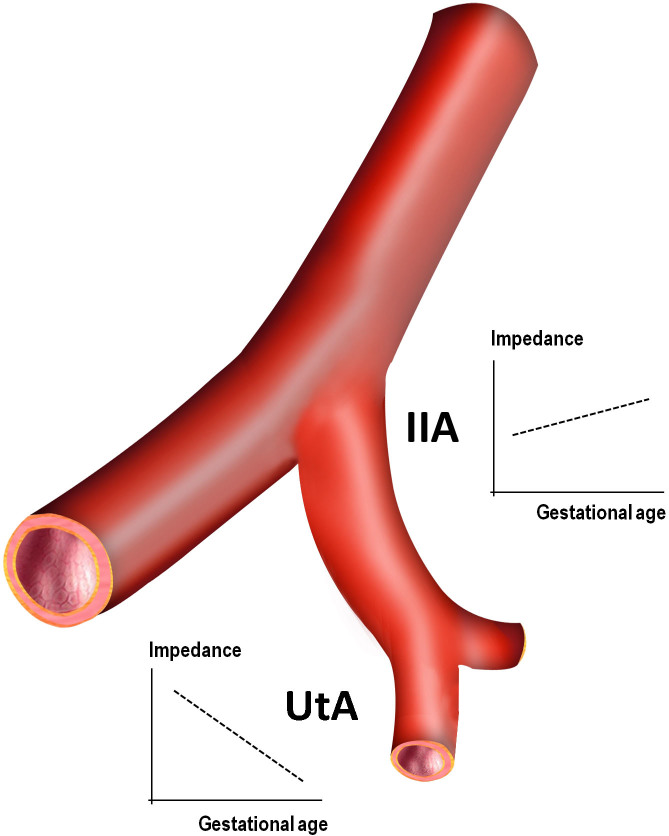
The internal iliac artery (IIA) precedes the uterine artery (UtA) but has a distinct impedance pattern along the pregnancy. Unlike the capacitance ability of the UtA, whose resistance reduces, the IIA exhibits a progressive increase.

These findings favour the view that along the pregnancy there is an adaptation of the mother circulation to the growing foetus so that the low resistance uterine artery is rapidly filled by the enhanced maternal cardiac output and flow velocity of the internal iliac artery (Figure 4). Moreover, the adaptation is progressive as the trimester-related upward shift of IIA to UtA proportion evidenced. We are convinced that this change provides the local circulation with a reserve capacity that meets the growing foetal needs.

The pregnancy condition also appears to allow the adaptability in another stable, yet different, hemodynamic condition as is the case of long term hypertensive, non-medicated women. They are known to be at risk for developing preeclampsia and intrauterine growth retardation, but coursed with an uneventful pregnancy and delivered a healthy infant.

The present study showed that the uterine and internal iliac arteries exhibit a performance that parallel the normotensive condition, although at a slightly different level of PI and RI. Therefore, a hemodynamic adaptation is likely to have occurred, probably even before pregnancy, as the 1^st^ trimester PI and RI values of both arteries already evidence. The relative changes established within PIs and RIs, show that in hypertensive pregnant women too, a high velocity flow coming from the internal iliac artery feeds the large capacity uterine arteries and placental intervillous space with nutrient and oxygen rich blood in a progressive, regular pattern.

From this comparison and the elimination of confounding variables, a model emerged indicating that perfusion of the pregnant uterus is not dependent on the mother's age, parity or hypertensive status. Rather, it appears to depend on pelvic circulation regulatory mechanisms that sense the local needs and adapt the arterial flow properties immediately upon entering the pelvis, endowing the internal iliac artery with a determinant role in uterine perfusion.

The specifics of these adaptive hemodynamic mechanisms and how they interact with the structural placental bed changes are unknown. Similarly, it is uncertain whether these findings, collected from stable conditions, are affected by risk factors known to lead to rapidly changing vascular features and pregnancy complications. The fact that the hypertensive condition puts the PIs and RIs of both arteries at different levels, suggests that their evaluation will be beneficial to high risk pregnancy assessment.

In summary, the distinct impedances of internal iliac and uterine here shown in normotensive and chronic hypertensive pregnant women are evidence of a resistance vessel that precedes a typical capacitance vessel. Moreover, apart from the novelty of the IIA data, the results and the developed model suggest a continuous local circulatory adaptation to meet the needs of the growing foetus. They also point to IIA as an accessible, interesting additional target for the prognostic study of obstetrical disorders with major vascular component.

In conclusion, the impedance of the internal iliac artery of normotensive and hypertensive women evidences a progressive increase along the pregnancy, which contrasts notably with the decreasing trend of the uterine artery. This variation adjusts to a model of a continuous filling of the uterine artery by an adaptive internal iliac artery, suggesting a local regulatory mechanism imparted by the pregnancy condition.

## Methods

### Subjects

The research protocol was approved by the local ethics committee [Ref. 133/10(086-DEFI/126-CES)] of Centro Hospitalar do Porto, Unidade Maternidade Júlio Dinis (CHP-MJD) and all subjects gave their informed consent upon adequate explanation.

From January 2010 and December 2012, a total of 152 pregnant caucasian women were recruited to the study employing as basic criteria to be healthy or to have stable chronic hypertension without known target organ involvement. They had been referred by their family doctors to the CHP-MJD according to local pregnancy health policies.

In the first appointment, that coincided with the first ultrasound evaluation, they were observed by a senior specialist who reviewed the patient's history, verified the absence of diabetes and other endocrine disorders, immune diseases, renal and structural heart diseases, haematological conditions and chronic infections; it was also checked the gestational age (GA) by sonography between 11 and 14 weeks and measured the blood pressure (BP). None of the women had a history of preeclampsia (PE) and only 1 reported having had hypertension during a previous pregnancy. Acceptable medication was folic acid, vitamin and iron supplements and acetylsalicylic acid, 100 mg per day, prescribed to all hypertensive women since the first appointment, until the last Doppler data collection.

Hypertension (HT) was defined as systolic BP ≥140 mmHg and/or diastolic BP ≥90 mmHg, present before pregnancy or the 20th week^26^,^27^,^28^,^29^. Mild to moderate hypertension in pregnancy was considered as systolic BP 140 to 159 or diastolic BP of 90 to 109 mm Hg, which corresponds closely to stage 1 of essential hypertension, defined as systolic 140 to 159 mmHg or diastolic 90 to 99 mm Hg^27^. An average of two BP measurements after a 4 hours period of rest was calculated.

All women were then enrolled in a longitudinal prospective study which included a trimestral ultrasound evaluation (centred at 13.04 ± 0.68, 20.73 ± 0.78 and 30.46 ± 1.19 weeks) and the recommended regular blood tests. Body Mass Index (BMI) was determined upon biometrical data collected at the hospital, before the first ultrasound evaluation.

Along the follow-up, a close attention was put on the appearance of abnormal conditions in the mother and foetus. These included foetal abnormal Doppler ultrasound indices in the umbilical and middle cerebral arteries, and foetal growth <10th and >90th percentile^30^,^31^. Moreover, as all women delivered at CHP-MJD, the healthy condition of the infant was verified by a neonatologist at birth and one month later.

In the follow-up, 49 women (32.2%) were excluded because of events occurring along the pregnancy. These were diabetes (n = 13), psychiatric disorders (n = 8), need of chronic medication beyond the established (n = 4), autoimmune disease (n = 4), later refusal to participate (n = 1), foetal/newborn pathology (n = 4) and failed sonographic evaluation at the defined schedule (n = 15).

Therefore, at the end, among the 103 women who were enrolled in the study, 72 were normotensive (NT) and 31 had chronic hypertension (HT).

### Doppler flow study

The Doppler flow study of both right and left internal iliac and uterine arteries was made immediately before the routine trimestral transabdominal obstetrical ultrasound scan employing a Voluson E8 or a Voluson 730 Pro (GE Healthcare Technologies, USA) device, equipped with multifrequency transvaginal and transabdominal transducers.

All measurements were performed by a single investigator with extensive experience in Doppler ultrasound (A.C.), in order to minimize inter-observer variability. Smokers were required to abstain from smoking for at least 2 h prior to examination.

For the exam, the probe was placed on the lower quadrants of the abdomen, angled medially and colour Doppler imaging was used to localize the UtA at its crossing over the external iliac artery. A minor movement of the probe towards the flanks, complemented with a slight medial rotation, evidenced the common iliac artery and its division. As soon as the internal iliac artery was identified, the measurement was made at right and left sides. The procedure became rather easy upon a brief period of training.

In all cases, after an angle less than 30° was assured and pulsed Doppler probe was placed over the whole vessel width, IIA measurements were collected next to the bifurcation of the common iliac artery (Figure 1A). Angle correction was then applied and the signal updated until three similar consecutive waveforms were evidenced, just before calculating left and right uterine arteries PI and RI, using the software of the device (Figure 1B).

The presence or absence of a bilateral early protodiastolic notch in UtA was noted (Figure 1C, D). A positive notch was defined as a persistent decrease in blood flow velocity in early diastole, below the diastolic peak velocity in at least one UtA Doppler ultrasound spectrum. According to the same reasoning, absence of notch was defined by its bilateral absence.

### Statistical analysis

Univariate data analysis comprised standard statistical methods: the chi-square test or the Fisher test (as adequate) for the study of independence amongst two factors, and the *t*-test for the assessment of statistically significant differences across means in two independent populations.

Multiple linear regression models with errors that were allowed to be correlated and/or to have unequal variances were fitted using generalized least squares. Multivariate regression had to be considered due to the experiment's nature: two different indexes were read on two different vessels for the same set of individuals, once at each trimester of the pregnancy. We looked for adequate global models and compared the curves (as functions of time but adjusted for potential confounders) instead of only doing comparison of mean indices between different time points.

The response variable read for index *d* (PI or RI), at vessel *v* (UtA or IIA), in a subject presenting notching at the first trimester with the status *s* (present or absent) and hypertension with the status *h* (hypertensive or normotensive), at (continuous) time *t* was denoted by *R(d,v,s,h,t)*. Dummy variables had to be considered for the index, vessel, status of notching at the first trimester and status of hypertension; reference categories were taken to be the resistance index, internal iliac artery, the normotensive status and the non-existence of unilateral notching at the first trimester, respectively. The fitted model was
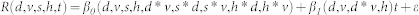
with residuals *ε* following a normal distribution with zero mean and with a variance-covariance matrix that allowed for a time autocorrelation structure of order 1 and for different variances across the indexes. In the above formula, the intercept coefficient *β_0_* is a function of the index, vessel, hypertensive status, unilateral notching status, and their two-way interactions, while the time-slope coefficient *β_1_* is a function of the index, vessel, and their interaction.

In order to understand the dynamic transition of each index (PI and RI) from the internal iliac artery to the uterine vessels, a model for the relative change was considered employing the quotient: (IIA index value – UtA index value)/(IIA index value). More precisely, for each of the indexes, the difference between its values on the internal iliac and uterine arteries was divided by the value read at the internal iliac artery for each trimester. Again a multiple regression model with correlated and heteroscedastic errors was adjusted, via generalized least squares.

Similarly to above, the response variable representing the proportion read for fixed index *d*, hypertensive status *h*, unilateral notching status *s* and time *t*, was denoted by *P(d,h,s,t)*. Dummy variables had to be considered for the index, status of unilateral notching at the first trimester and the status of hypertension; reference categories were taken to be the resistance index, the normotensive status and the non-existence of unilateral notching at the first trimester, respectively. The fitted model was
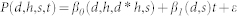
with an intercept coefficient *β_0_* depending on *d*, *h*, *s* and the interaction term d*h, a time-slope coefficient depending on d and s, and residuals *ε* following a normal distribution with zero mean and with a variance-covariance matrix that allowed for a time autocorrelation structure of order 1 and for different variances across the indexes.

Final regression models were chosen on the basis of the lowest BIC (Bayesian Information Criterion). All statistical analyses were carried out using the R language and software environment for statistical computation, version 2.12.1^32^. The significance level was fixed at 0.05, as usual.

## Author Contributions

L.G.-M. and H.A. designed the study, analyzed the data and wrote the manuscript; A.C. coordinated quality control of ultrasound data; J.S. coordinated review of clinical cases and organization of study groups; R.G. performed all statistical analyses; F.M. designed the study. All authors contributed to the data interpretation and the final version of the manuscript, which they all approve.

## Figures and Tables

**Table 1:** Main characteristics and pregnancy outcomes of 103 women included in the study

		n (%)	p-value^2^	Normotensive (NT, n = 72)	Hypertensive (HT, n = 31)	p-value^3^
Age (intervals in years)	17–24	13 (13%)	<0.001	13 (18%)	0 (0%)	<0.001
	25–34	58 (56%)		46 (64%)	12 (39%)	
	35–43	32 (31%)		13 (18%)	19 (61%)	
Education level (in years)	<7	5 (5%)	<0.001	3 (4%)	2 (6%)	0.579
	7–9	31 (30%)		23 (32%)	8 (26%)	
	10–12	38 (37%)		24 (33%)	14 (45%)	
	>12	29 (28%)		22 (31%)	7 (23%)	
Smoking	No	84 (82%)	<0.001	61 (85%)	23 (74%)	0.308
	Yes	19 (18%)		11 (15%)	8 (26%)	
Parity	0	54 (52%)	0.556	43 (60%)	11 (35%)	0.036
	≥1	49 (48%)		29 (40%)	20 (65%)	
Body Mass Index^1^ (Kg/m^2^)	18–24	56 (54%)	<0.001	50 (69%)	6 (19%)	<0.001
	25–29	32 (31%)		15 (21%)	17 (55%)	
	30–51	15 (15%)		7 (10%)	8 (26%)	
Age at menarche	12.5 (1.6)		–	12.4 (1.5)	12.8 (1.8)	0.243
Age at first intercourse	18.1 (2.0)		–	17.9 (1.8)	18.5 (2.3)	0.205
Trimestral evaluation (weeks ± SD)	13.04 (0.68)		<0.001	13.13 (0.73)	12.82 (0.51)	0.012
	20.73 (0.78)			20.79 (0.80)	20.59 (0.71)	0.210
	30.46 (1.19)			30.61 (0.74)	30.11 (1.85)	0.158
GA at delivery (weeks ± SD)	38.9 (1.7)		–	38.9 (1.65)	38.9 (1.68)	0.956

^1^BMI: measurement in trimester 1;

^2^p - tests equality of population frequencies amongst the different categories of a variable;

^3^p - tests homogeneity of the proportions between HT (hypertensive) and NT (normotensive). SD: standard deviation.

**Table 2:** Absolute (relative, %) frequencies for positive notching of uterine arteries along the pregnancy (n = 103), in normotensive (n = 72) and hypertensive (n = 31) women at each trimester

	n (%)	p-value^1^	Normotensive (n = 72)	Hypertensive (n = 31)	p-value^2^
Trimester 1	49 (48)	0.695	41 (57)	8 (26)	0.006
Trimester 2	16 (16)	<0.001	12 (17)	4 (13)	0.772
Trimester 3	5 (5)	<0.001	5 (7)	0 (0)	0.319

^1^p - tests equality of population frequencies amongst positive and negative notching;

^2^p - tests homogeneity of the proportions between HT and NT.

**Table 3:** Mean (SD) uterine and internal iliac artery PI and RI indices, measured by transabdominal ultrasound examination at the different trimesters

	IIA			UtA		
	Trimester			Trimester		
	1	2	3	1	2	3
	NT (n = 72)					
PI	2.87 (0.50)	3.16 (0.53)	3.30 (0.63)	1.56 (0.50)	0.99 (0.30)	0.79 (0.18)
RI	0.88 (0.06)	0.90 (0.04)	0.91 (0.05)	0.70 (0.11)	0.57 (0.08)	0.50 (0.07)
	HT (n = 31)					
PI	2.68 (0.40)	2.69 (0.33)	3.08 (0.33)	1.35 (0.41)	1.05 (0.27)	0.84 (0.12)
RI	0.88 (0.04)	0.88 (0.04)	0.93 (0.04)	0.63 (0.07)	0.57 (0.08)	0.48 (0.07)

Abbreviations: IIA, internal iliac artery; UtA, uterine artery; NT, normotensive; HT, hypertensive; PI, pulsatility index; RI, resistance index.

**Table 4:** Estimated coefficients and 95% confidence intervals (CI) of the regression model used to obtain the expected indices at the different covariates combinations

Covariates	Coefficient	95% CI
Intercept	0.860	(0.836, 0.884)
PI	1.705	(1.576, 1.834)
UtA	−0.108	(−0.135, −0.081)
Hypertension	−0.026	(−0.057, 0.005)
Bilateral Notching	0.023	(0.009, 0.037)
PI * UtA	−0.791	(−0.954, −0.628)
Bilateral Notching * PI	0.190	(0.123, 0.257)
Bilateral Notching * UtA	0.036	(0.016, 0.056)
Hypertension * PI	−0.219	(−0.319, −0.119)
Hypertension * UtA	−0.016	(−0.038, 0.006)
Hypertension * PI * UtA	0.287	(0.158, 0.416)
Time	0.012	(0.003, 0.021)
Time * PI	0.197	(0.142, 0.252)
Time * UtA	−0.109	(−0.121, −0.097)
Time * PI * UtA	−0.449	(−0.522, −0.376)
Time * Hypertension	0.015	(0.001, 0.029)

Abbreviations: UtA, uterine artery; NT, normotensive; PI, pulsatility index.

**Table 5:** Estimated coefficients and 95% confidence intervals (CI) of the regression model for the expected relative changes of RI and PI

Covariates	Coefficient	95% CI
Intercept	0.199	(0.156, 0.242)
PI	0.229	(0.196, 0.262)
Hypertension	0.005	(−0.020, 0.030)
Bilateral Notching	−0.176	(−0.227, −0.125)
PI * Hypertension	−0.049	(−0.076, −0.022)
Time	0.087	(0.067, 0.101)
Time * PI	0.029	(0.013, 0.045)
Time * Notching	0.059	(0.035, 0.083)

Abbreviations: PI, pulsatility index; CI, confidence intervals.
